# Comparison of COVID-19 and RSV Infection Courses in Infants and Children under 36 Months Hospitalized in Paediatric Department in Fall and Winter Season 2021/2022

**DOI:** 10.3390/jcm11237088

**Published:** 2022-11-29

**Authors:** Anna Fedorczak, Natalia Zielińska, Paulina Nosek-Wasilewska, Katarzyna Mikołajczyk, Joanna Lisiak, Krzysztof Zeman, Marcin Tkaczyk

**Affiliations:** 1 Department of Pediatrics, Nephrology and Immunology, Medical University of Lodz, 90-419 Lodz, Poland; 2 Department of Endocrinology and Metabolic Diseases, Polish Mother’s Memorial Hospital Research Institute, 93-338 Lodz, Poland; 3 Department of Pediatrics, Immunology and Nephrology, Polish Mother’s Memorial Hospital Research Institute, 93-338 Lodz, Poland

**Keywords:** COVID-19, SARS-CoV-2, RSV, pediatrics, children, infants, outcome

## Abstract

Background: The study aimed to determine the differences between COVID-19 and Respiratory syncytial virus (RSV) infections in young children hospitalized in the pediatric department. Methods: This retrospective study included 52 children with COVID-19 and 43 children with RSV infection younger than 36 months hospitalized in a pediatric department between September 2021 and March 2022. Clinical and laboratory findings, methods of treatment and hospitalization length were compared. Results: In the RSV group, significantly higher rates of cough (93.2% vs. 38.5%), rhinitis (83.7% vs. 50%), dyspnea (83.7% vs. 21.1%), crackles (69.8% vs. 5.8%) and wheezes (72.1% vs. 9.6%) were observed. The COVID-19 group had significantly higher rates of fever (80.8% vs. 37.2%) and seizures (13.5% vs. 0%). Patients with RSV infection had significantly higher rates of bronchodilator therapy (88.37% vs. 5.77%) and oxygen therapy (48.8% vs. 7.7%) and required a longer hospital stay (8 vs. 3 days). In admission, the majority of the patients from both groups were not treated with antibiotics, but because of clinical deterioration and suspected bacterial co-infections, antibiotics were administered significantly more frequently in the RSV group (30.2% vs. 9.6%). Conclusions: RSV infection in infants and small children had a more severe course than COVID-19 infection. RSV infection was associated with a longer hospitalization period and required more elaborate treatment.

## 1. Introduction

The coronavirus disease (COVID-19) is caused by the severe acute respiratory syndrome coronavirus 2 (SARS-CoV-2), which was firstly identified in December 2019 in Wuhan and spread worldwide, leading to a global pandemic. In total, there have been 616,951,418 confirmed cases and 6,530,281 deaths reported to WHO as of 6 October 2022 [1]. It manifests mainly as respiratory tract infections, which present from mild to severe, but other symptoms and complications are also reported. The pediatric population is less affected by SARS-CoV-2; children and adolescents under 20 years of age account for 21% of the reported COVID-19 and 0.4% of total deaths [2,3]. According to the United Nations International Children’s Emergency Fund (UNICEF) data, by September 2022, children aged 0–4 years represent 3.9% of all reported COVID-19 cases and 0.1% of deaths [3]. Children have milder symptoms, better prognoses and lower mortality rates than adults [4]. Nevertheless, the course of COVID-19 in children varies, and some of the affected patients may present severe illness and require hospital admission and specialized treatment [5]. Moreover, in some cases, after SARS-CoV-2 exposure or infection, multisystem inflammatory syndrome in children (MIS-C) may occur [6].

Respiratory syncytial virus (RSV) is a common cause of respiratory tract infection that affects people of all ages. RSV disease in children manifests from mild upper respiratory tract infection to severe lower respiratory tract infection, which may require hospitalization and lead to serious complications such as respiratory failure [7]. Infants and young children are at risk of the severe course of the disease. RSV is the major cause of hospital admissions in young children [7]. Shi et al. estimated that globally, in 2015, 33.1 million episodes of RSV respiratory infection cases resulted in about 3.2 million hospital admissions and 59,600 in-hospital deaths in children younger than 5 years [8]. 

A review of the literature indicates that a limited number of studies have compared the clinical picture of these two diseases in young children [9,10]. Both retrospective analyses of the local cohorts of neonates and young children under 4 years old showed that RSV-infected patients required a higher level of medical care and had to stay in hospital longer than SARS-CoV-2–infected children. The study aimed to determine the differences between COVID-19 and RSV infection courses in infants and young children hospitalized in the pediatric department in the winter season of 2021/2022.

## 2. Materials and Methods

This retrospective study included 52 children with COVID-19 and 43 children with RSV infection younger than 36 months hospitalized in a pediatric department in Lodz, Poland, between September 2021 and March 2022. This time period was chosen because in the previous season of 2020/2021, in relation to the COVID-19 pandemic, there were no hospitalized patients aged 0–3 years with RSV infection in our department. 

RSV was detected by the Acro Biotech rapid antigen test. The rapid antigen test (Panbio™ COVID-19 Ag Rapid Test Device) was used for the detection of SARS-CoV-2 antigen. Presence of SARS-CoV-2 was confirmed by real-time PCR test. Inclusion criteria assumed: 1. Positive viral PCR for SARS-CoV-2 or antigen test for RSV; 2. Hospital admission from 1 September 2021 to 31 March 2022; 3. Age 0–36 months. 4. Symptoms of the respiratory tract infection. Patients positively tested with SARS-CoV-2 who were asymptomatic and hospitalized in the COVID unit of the pediatric department because of different reasons (e.g., fracture) were excluded from the study. Patients infected with both SARS-CoV-2 and RS Vor with other infections (f.e. rotavirus) were excluded from the analysis. Clinical and laboratory findings, methods of treatment and hospitalization length were compared. 

The statistical analysis of collected data was performed using STATISTICA ver. 13.3 software (Statsoft, Kraków, Poland). The Shapiro–Wilk test was used for the assessment of normal distribution and Levene’s test for the equality of variances. Between-group continuous variable comparisons were performed with non-parametric Mann–Whitney’s U test. Between-group nominal variables comparisons were performed with Chi-square test, and Yates’ correction was used where eligible. Continuous variables were summarized with median and interquartile ranges (median (Q1–Q3)) and categorical variables with N (%)). A *p*-value < 0.05 was considered statistically significant.

The Bioethics Committee at the Medical University of Lodz was informed about this retrospective study. As this retrospective study did not have the characteristics of a medical experiment or clinical trial carried out on a patient, approval was not required.

## 3. Results

### 3.1. Study Group Characteristics and Baseline Clinical Features 

There were 52 children with COVID-19 and 43 children with RSV included in the study. The median age of patients with RSV infection was 4 months (Q1:Q3: 1–10), and for patients with COVID-19, 8.5 months (Q1–Q3: 4–14). A total of 24 (55.1%) patients with RSV infection were boys and 19 (44.19%) were girls, and 27 (51.92%) patients with COVID-19 were boys and 25 (48.08%) were girls. There were no statistically significant differences between compared groups with respect to age and sex (*p* = 0.35 and *p* = 0.7, respectively). The median time from the first symptoms to hospital admission for the RSV group was 3 days (Q1–Q3: 2:5) and for the COVID-19 group was 2 days (Q1–Q3: 1–3), *p* = 0.53. 

On admission, the most commonly presented symptom for COVID-19 group was fever (76.92%) and for the RSV group, cough (93.2%), dyspnea (76.74%) and rhinitis (72.09%). RSV patients presented with auscultatory changes (93.02% vs. 21.15%, *p* = 0.00) significantly more often. The baseline clinical features of RSV and COVID-19 patients at hospital admission are presented in Table 1.

### 3.2. Hospital Admission in the Present and Previous Seasons

**Figure 1:**
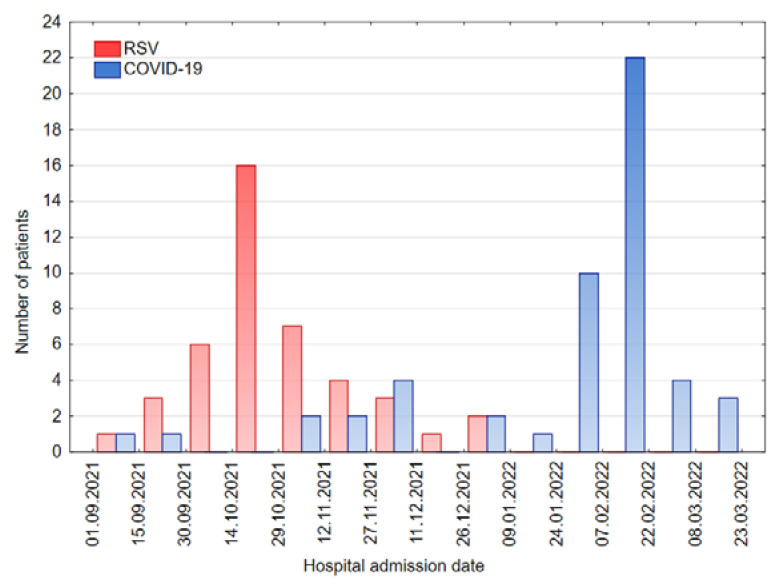
Weekly hospital admissions of patients with COVID-19 and RSV in the winter season of 2021/2022.

In the analyzed time period (between 1 September 2021 and 31 March 2022), patients diagnosed with RSV infection were admitted to the hospital between 1 September 2021 and 26 December 2021, whereas patients diagnosed with COVD-19 were mostly admitted between 12 November 2021 and 23 March 2022. Precise data are presented in the bar chart in Figure 1. 

In total, 43 patients with RSV infection were hospitalized in the peadiatric department in fall and winter season 2021/2022. By contrast, in the season 2020/2021, there were no patients aged 0–36 months hospitalized with RSV infection. The numbers of RSV-diagnosed patients hospitalized in the pediatric department in the previous seasons are presented in Table 2.

### 3.3. Frequency of Symptoms Presented by Patients with COVID-19 and RSV Infection during the Whole Hospital Stay

In the RSV group, significantly higher rates of cough (93.2% vs. 38.5%, *p* = 0.000), rhinitis (83.7% vs. 50%, *p* = 0.001), and dyspnea (83.7% vs. 21.1%, *p* = 0 000) were observed. On physical examination, the majority of patients with RSV infection had auscultatory changes (97.67% vs. 34.62%, *p* = 0.000): wheezes (72.1% vs. 9.6%, *p =* 0.000), crackles (69.8% vs. 5.8%, *p* = 0.000) and rhonchi (60.47% vs. 32.69% *p* = 0.007) were more often noticed. 

The COVID-19 group had significantly higher rates of fever (80.8% vs. 37.2%) and seizures (13.5% vs. 0%). Regarding nausea, lack of appetite, vomiting or diarrhea, there was no significant difference between the groups. 

### 3.4. Laboratory Findings

In the RSV group, oxygen saturation (Sp02) on admission (97 (Q1–Q3: 95–98) vs. 98 (Q1–Q3: 97–98,5), *p*-value 0.04) and during hospital stay (94 (Q1–Q3: 89–97) vs. 97 (Q1–Q3: 95–98,5), *p*-value 0.000) were significantly lower than in COVID-19 group. Moreover, partial pressure of carbon dioxide (pC02) was significantly higher and acidosis was more frequently found in the RSV group (36.59% vs. 12.5%).

In patients with COVID-19, median lymphocyte count was lower (4.19 × 10^3^/ul (Q1–Q3: 2.48–6.49) vs. 5.56 × 10^3^/ul (Q1–Q3: 5.56–7.1), *p* = 0.007) and lymphopenia more frequently observed (34.62% vs. 6.98%, *p* = 0.001). The neutrophil-to-lymphocyte ratio (NLR) was significantly higher than in the RSV group (0.81 (0.34–1.73 vs. 0.4 (0.26–0.79, *p* = 0.001))).

There was no statistically significant difference in the white blood cell (WBC) count, neutrophil count and C-reactive protein (CRP) levels between the groups. The procalcitonin (PCT) level was low in the vast majority of patients from both groups, but it was not measured in all of the patients, so the comparison could not be relevant.

### 3.5. Methods of Treatment

Most COVID-19 patients were given only saline inhalations or/and antipyretic drugs when needed (63.46% vs. 2.33%). Patients with RSV infection had significantly higher rates of bronchodilator therapy (88.37% vs. 5.77%) and passive oxygen therapy (48.8% vs. 7.7%).

In admission, there was no significant difference in the inhaled steroid therapy use, but during the hospital stay, it was introduced three times more often in patients with RSV infection. There was no significant difference in the frequency of systemic steroid therapy. In admission, the majority of the patients from both groups were not treated with antibiotics (RSV 86% and COVID-19 98.1%), but during the hospital stay, antibiotics were administered more often in the RSV group (30.2% vs. 9.6%). A summary of used treatment methods is presented in Table 3.

**Figure 2:**
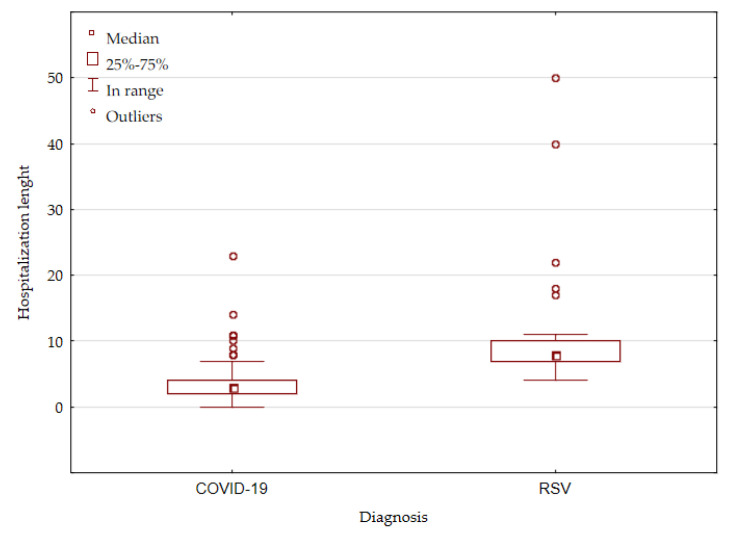
Hospitalization length comparison between the groups.

Individual patients from both groups required high flow nasal cannula (HFNC) oxygen therapy and intensive care unit (ICU) stay, but there was no statistically significant difference between the groups. All patients recovered from the illness. None of the hospitalized COVID-19 patients developed multisystem inflammatory syndrome in children (MIS-C). Patients with RSV infection required a significantly longer hospital stay (8 days (Q1–Q3: 7–10) vs. 3 days (Q1–Q3: 2–4), Figure 2).

## 4. Discussion

Respiratory syncytial virus (RSV) is a seasonal virus that typically peaks in fall and declines by early spring. In Poland and other countries of the northern hemisphere, these infections usually occur between October and May, with a peak in January–February [11]. The emergence of SARS-CoV-2 was associated with substantial reductions in the circulation of seasonal respiratory viruses. In 2020–2021, a significant change was observed in the epidemiology of respiratory syncytial virus infections in Europe and worldwide [12,13,14]. RSV circulation was nearly absent during the winter and appeared in spring. SARS-CoV-2 appears to be superior to other respiratory viruses in terms of resistance and infectivity [14]. Observations in surveillance systems indicate that RSV circulation increased during the summer and fall of 2021 (Delta predominance) and declined during the winter of 2021/2022 (Omicron predominance) [15]. The SARS-CoV-2 Omicron variant has been the predominant circulating variant in the United States since late December 2021 [16]. Correspondingly with the increase in the Omicron circulation, COVID-19-related hospitalization rates rose sharply among infants and children aged 0–4 years, a group that cannot yet be vaccinated [16].

Our results reflect similar findings: most RSV patients were admitted to hospital between September and November 2021, with the peak in mid-October, and a further decrease and subsequent disappearance at the end of the year, when the number of admitted COVID-19 patients was rising. Correspondingly, admissions of COVID-19 patients aged 0–3 years escalated from the beginning of 2022, with the peak in February during Omicron predominance (Figure 1). Backtracking to the previous winter seasons, in the 2020/2021 season, with the COVID-19 presence and all the pandemic-related restrictions, there were no RSV-infected patients under 36 months hospitalized in our pediatric department, whereas in 2019/2020 and 2018/2019, there were 56 and 35 admissions, respectively.

Factors that might have had an impact on the RSV circulation pattern change during the pandemic were strict public health measures, social distancing, and hygiene precautions, such as hand hygiene and mandatory face masks. Furthermore, the low rate of co-infections between SARS-CoV-2 and other respiratory viruses may suggest an interference between viruses and SARS-CoV-2 predominance [17]. Referring to our data, in the analyzed period, there were only two patients hospitalized with COVID-19 and RSV confection, and they were excluded from the analysis. However, Alvares et al. managed to compare patients with RSV and COVID-19 co-infections revealing that they required a longer hospital stay [18].

Conclusively, clinicians should be aware that respiratory viruses may not present typical seasonal circulation patterns, and the hospital background (adequate number of beds, oxygen stations, and special equipment) should be prepared throughout the year.

Both diseases may share similar features, and it is difficult to differentiate the infection cause on the basis of presented symptoms. As far as the relevant test is available, it should be conducted to clarify the diagnosis. Nevertheless, according to our research, the appearance of cough, dyspnoea or auscultatory changes may suggest that the RSV virus is a more likely cause of the infection. By contrast, patients who presented with fever were more often diagnosed with COVID-19. Our findings reflect the systematic review from 2021, which suggest that fever is the most common symptom of COVID-19-infected children, and most cases had mild-to-moderate disease severity [19]. In an RSV and SARS-CoV-2 infections comparison study conducted by Mayer et al., RSV patients also presented significantly more often with a cough, whereas SARS-CoV-2 patients suffered significantly more often from fever and gastrointestinal symptoms [10].

Multiple reports indicate that children with COVID-19 may present with neurological symptoms involving both the central and peripheral nervous systems [20,21]. A great variety of symptoms have been reported from mild symptoms, such as headache and anosmia, to severe manifestations, such as stroke, seizure or encephalopathy. In our research, the only neurological symptoms presented by children with COVID-19 were seizures, which were observed in 13.5% of patients, while there was no seizure occurrence in the RSV group. During that time period in our hospital, some more serious neurological complications of COVID-19, such as stroke, were observed but did not occur in the analyzed age group of patients. Therefore, clinicians should be aware of the potential neurological consequences of COVID-19 to provide the patient with the best care.

Regarding laboratory findings, as both infections are caused by viruses, inflammatory markers in admission were predominantly low. Prozan et al. showed that COVID-19 adult patients had a lower NLR in admission than RSV patients. High NLR was identified to be a prognostic factor for poor clinical outcome [22]. In our research, NLR was higher in the COVID-19 group, but it did not reflect the severity of the disease. This may be in relation to lower lymphocyte count and lymphocytopenia that were reported more often in COVID-19 patients. There was only one patient with high NLR but mild symptoms. In both groups, there was no correlation between NLR level and length of hospital stay. Acidosis, which was more often found in patients with RSV infection, corresponded with their oxygen saturation and clinical manifestation with dyspnea and auscultatory changes due to lower respiratory tract obstruction and inflammation.

COVID-19 guidance for the management of children admitted to hospital indicates that all patients should be provided with supportive care, whereas bronchodilators should not be used routinely unless there is suspicion of bronchoconstriction. In the age group between 44 weeks gestational age and 5 years, corticosteroids should be considered for patients with severe or critical COVID-19. The antibiotic should be administered according to normal practice, taking into account the risk of sepsis and the fact that an alternative diagnosis and focus of infection has been investigated [23].

According to NICE guidelines, Remdesivir should be considered for hospitalized children over 12 years and 40 kg with COVID-19 that require supplemental oxygen or do not need supplemental oxygen for COVID-19 but are within 7 days of symptom onset and are thought to be at high risk of progression to severe COVID-19 [24]. Remdesivir use in younger patients should be in the context of a clinical trial or can be considered on a case-by-case basis after discussion with pediatric infectious diseases specialists [23,24].

In our study, all COVID-19 patients were provided with supportive care, 63% of whom, because of mild or moderate symptoms, did not require any other methods of treatment. During the entire hospital stay, they were given only saline inhalations or/and antipyretic drugs when needed. In several cases, oxygen supply, inhaled or intravenous corticosteroids, bronchodilators and antibiotic therapy were introduced, whereas Remdesivir was not available in hospital, and the indications in this age group were not clear.

By contrast, the vast majority of patients with RSV infection, besides supportive care, required more elaborate treatment. The reason for the use of bronchodilators, which were administered in most of the patients, was dyspnea and wheezes ascertained on physical examination. In cases of severe clinical course with increasing dyspnea and higher oxygen supply needs, inhaled and intravenous corticosteroids were introduced.

Treatment of RSV infection remains a major clinical issue. Therapy of RSV infection is mainly supportive, and only oxygen therapy, mechanical ventilation and fluid therapy are proven to be effective [25,26]. The clinical course of bronchiolitis resembles an exacerbation of asthma, hence the frequent off-label use of bronchodilators and inhaled and intravenous corticosteroids [11]. Antibiotics are not recommended for bronchiolitis unless there is concern about complications such as secondary bacterial pneumonia or respiratory failure. Nevertheless, they are often used [27].

Referring to our study, in admission, the majority of patients from both groups were not treated with antibiotics. However, they were administered in some cases and more often in the RSV group. The reason for antibiotic therapy was clinical deterioration and suspected or proven bacterial confection (determined by the intensification of auscultatory changes, dyspnea, saturation deterioration, laboratory inflammation indicators increase, lung ultrasonography or chest x-ray changes).

All patients recovered from the illness. Suspected bacterial supra-infection was the most often observed short-term complication. RSV-infected patients required more elaborate treatment and had to stay in hospital longer than SARS-CoV-2–infected children, thereby placing a greater strain on hospital resources. The differences in high-flow nasal cannula oxygen therapy and admission to the intensive care unit between the groups were not observed, but the study group was relatively small.

Mayer et al. concluded, in the retrospective study conducted in Germany where 169 RSV-infected and 24 SARS-CoV-2-infected children aged 0–4 were compared reached similar conclusions, that symptomatic patients with RSV were sicker than patients with SARS-CoV-2, required a higher level of medical care and were hospitalized significantly longer. Moreover, their findings showed that patients with RSV infection had a higher need for respiratory support with HFNC and had a higher risk of intensive care treatment, nCPAP and intubation [10].

Although the outcome of both infections was good, further follow-up is needed to assess the risk of potential long-term consequences of infection among infants and young children. Long-term complications after RSV infections, such as wheezing during the preschool years, risk of asthma or repeated respiratory infections, should not be forgotten [28,29]. Data indicate that COVID-19 may lead to long-term consequences as well. People with COVID-19 might have sustained postinfection sequelae. Common symptoms that include fatigue, shortness of breath, and cognitive dysfunction have an impact on everyday functioning [30]. One-quarter of every 10 children experience multisystem involvement [31]. It is also important to be aware of the potential low risk of developing multisystem inflammatory syndrome in children (MIS-C), a rare but severe hyperinflammatory systemic reaction with fever, hypotension, cardiac dysfunctions and other organ involvement, which may occur a few weeks after symptomatic or asymptomatic SARS-CoV-2 infection and mostly affects older children, with a median age of 6–11 years old [32,33]. Further studies are vital to understanding the possible long-term complications of COVID-19 in infants and young children.

## 5. Conclusions

In conclusion, both diseases may share similar features that may cause some difficulties in initial diagnosis, but cough, rhinitis and auscultatory changes are more frequent in RSV patients, while fever and seizures are more frequent in COVID-19 patients. Acidosis appears more often in RSV infection patients and lymphopenia in COVID-19 patients. RSV infection in infants and small children had a more severe course than COVID-19. RSV infection was associated with a longer hospitalization period and required more elaborate treatment. Since the appearance of the SARS-CoV-2 virus, the respiratory syncytial virus has not presented typical seasonal circulation patterns; therefore, clinicians should be prepared to provide infected patients with the best care throughout the year.

## 6. Study Limitations

Our study data were retrospectively collected from the medical records of patients hospitalized only in one department. The number of patients in both groups is small, and data are limited. However, our results show statistically significant differences that seem to be interesting. It may be reasonable to conduct a prospective, multicenter study in the following seasons.

## Figures and Tables

**Table 1:** The baseline clinical features of RSV and COVID-19 patients at hospital admission.

Baseline Clinical Features	COVID-19	RSV Infection	*p*-Value
cough	20 38.50%	40 93.20%	0.000
rhinitis	11 21.15%	31 72.09%	0.000
fever	40 76.92%	16 37.21%	0.003
dyspnea	10 19.23%	33 76.74%	0.000
weakness	15 28.85%	13 30.23%	0.882
lack of appetite	23 44.23%	18 41.86%	0.816
vomiting or diarrhea	12 23.08%	5 11.63%	0.552
seizures	7 13.46%	0 0.00%	0.035
auscultatory changes	11 21.15%	40 93.02%	0.000
wheezes	2 3.85%	29 67.44%	0.000
crackles	2 3.85%	28 65.12%	0.000
rhonchi	10 19.23%	18 41.86%	0.122
stridor	5 9.62%	0 0.00%	0.100

**Table 2:** Number of patients with RSV infection hospitalized in the fall and winter seasons of 2018–2022.

Fall and Winter Season	Number of RSV-Diagnosed Patients
2021/2022	43
2020/2021	0
2019/2020	56
2018/2019	35

**Table 3:** Treatment methods used in patients with COVID-19 and RSV infection.

Treatment Methods	COVID-19	RSV Infection	*p*-Value
bronchodilator therapy	3 5.77%	38 88.37%	0.000
inhaled steroid therapy in admission	8 15.38%	14 32.56%	0.083
inhaled steroid therapy in total	12 23.08%	26 60.47%	0.000
systemic steroid therapy	6 11.54%	11 25.58%	0.065
passive oxygen therapy	4 7.69%	21 48.84%	0.000
high flow nasal cannula oxygen therapy	1 1.92%	4 9.30%	0.254
antibiotic therapy in admission	1 1.92%	6 13.95%	0.065
antibiotic therapy in total	6 11.54%	19 44.19%	0.001
supportive care only	33 63.46%	1 2.33%	0.000

## Data Availability

The data presented in this study are available on request from the corresponding author.
